# Altered Affinity Maturation in Primary Response to (4-hydroxy-3-nitrophenyl) Acetyl (NP) after Autologous Reconstitution of Irradiated C57BL/6 Mice

**DOI:** 10.1080/1044667031000137593

**Published:** 2002-09

**Authors:** Carl De Trez, Annette Van Acker, Georgette Vansanten, Jacques Urbain, Maryse Brait

**Affiliations:** Universite Libre de Bruxelles Institut de Biologie et de Médecine Moléculaires Laboratory of Animal Physiology 12 rue des Profs Jeener et Brachet Gosselies 6041 Belgium

## Abstract

Immune responses developing in irradiated environment are profoundly altered. The memory anti-arsonate response of A/J mice is dominated by a major clonotype encoded by a single gene segment combination called CRIA. In irradiated and autoreconstituted A/J mice, the level of anti-ARS antibodies upon secondary immunization is normal but devoid of CRIA antibodies. The affinity maturation process and the somatic mutation frequency are reduced. Isotype switching and development of germinal centers (GC) are delayed.

The primary antibody response of C57BL/6 mice to the hapten (4-hydroxy-3-nitrophenyl) acetyl (NP)-Keyhole Limpet Hemocyanin (KLH) is dominated by antibodies encoded by a family of closely related VH genes associated with the expression of the λ1 light chain.We investigated the anti-NP primary response in irradiated and autoreconstituted C57BL/6 mice. We observed some splenic alterations as previously described in the irradiated A/J model. Germinal center reaction is delayed although the extrafollicular foci appearance is unchanged. Irradiated C57BL/6 mice are able to mount a primary anti-NP response dominated by λ1 positive antibodies but fail to produce high affinity NP-binding IgGl antibodies. Following a second antigenic challenge, irradiated mice develop enlarged GC and foci. Furthermore, higher affinity NP-binding IgG1 antibodies are detected.


*Developmental Immunology*, September 2002, Vol. 9 (3), pp. 119–125

Taylor & Francis

Taylor & Francis Group

### Altered Affinity Maturation in Primary Response to (4-hydroxy-3-nitrophenyl) Acetyl (NP) after Autologous Reconstitution of Irradiated C57BL/6 Mice*

CARL DE TREZ, ANNETTE VAN ACKER, GEORGETTE VANSANTEN, JACQUES URBAIN and MARYSE BRAIT[†]

*Universite Libre de Bruxelles, Institut de Biologie et de Médecine Moléculaires, Laboratory of Animal Physiology, 12 rue des Profs Jeener et Brachet, Gosselies 6041, Belgium*

Immune responses developing in irradiated environment are profoundly altered. The memory anti-arsonate response of A/J mice is dominated by a major clonotype encoded by a single gene segment combination called CRIA. In irradiated and autoreconstituted A/J mice, the level of anti-ARS antibodies upon secondary immunization is normal but devoid of CRIA antibodies. The affinity maturation process and the somatic mutation frequency are reduced. Isotype switching and development of germinal centers (GC) are delayed.

The primary antibody response of C57BL/6 mice to the hapten (4-hydroxy-3-nitrophenyl) acetyl (NP)-Keyhole Limpet Hemocyanin (KLH) is dominated by antibodies encoded by a family of closely related VH genes associated with the expression of the λ1 light chain.We investigated the anti-NP primary response in irradiated and autoreconstituted C57BL/6 mice. We observed some splenic alterations as previously described in the irradiated A/J model. Germinal center reaction is delayed although the extrafollicular foci appearance is unchanged. Irradiated C57BL/6 mice are able to mount a primary anti-NP response dominated by λ1 positive antibodies but fail to produce high affinity NP-binding IgGl antibodies. Following a second antigenic challenge, irradiated mice develop enlarged GC and foci. Furthermore, higher affinity NP-binding IgG1 antibodies are detected.

*Keywords*: Germinal centers; Affinity maturation; Irradiation; NP system; Lymphopenic environment

### INTRODUCTION

The studies of anti-hapten antibody responses show that humoral immune responses are dynamic processes where extensive changes in both clonal composition of the responding B cell population and the structure and function of the antibodies expressed take place. The response to the arsonate hapten coupled to Keyhole Limpet Hemocyanin (KLH) has been well characterized in A/J mice. Early after a primary immunization with ARS-KLH, A/J mice produce anti-arsonate antibodies encoded by several genetic combinations but as the response proceeds, a major canonical combination, namely the CRIA idiotype, emerges and dominates the memory responses (Manser *et al.*, 1987; Rathbun *et al.*, 1988). This single canonical combination encoded by VHIDCR11-DFL16.2-JH2/VK10-JK1 is subjected to a process of hypermutation, allowing the generation and selection of variants of higher affinity for antigen. The idiotype CRIA is therefore, a good marker of anti-arsonate memory response. Immunohistochemical analyses indicate that splenic ARS + CRIA + germinal centers (GC) and plasma cells are easily detected during the secondary response in contrast to the primary response (Vora *et al.*, 1998; 1999; our unpublished observations).

In the course of transfer experiments performed in our laboratory, we fortuitously discovered that the CRIA idiotype expression was profoundly altered when the anti-ARS response took place in an irradiated environment. For instance we analyzed irradiated A/J mice (650 rads) reconstituted with either syngeneic naive spleen cells or bone marrow cells or irradiated with their hind limbs partially shielded allowing autoreconstitution. Some hallmark of memory is retained in these irradiated recipients: higher concentration of antibodies in the secondary response. But other hallmarks are lost: the CRIA idiotype expression and the affinity maturation (Willems *et al.*, 1990). Moreover, the isotype switching and the development of GC are delayed. Molecular analysis performed on a set of anti-ARS monoclonal

*Presented at the Proceedings of the 4th Germinal Center Conference, Groningen, The Netherlands, June 2002.

[†]Corresponding author. Tel.: +32-2-650-98-65. Fax: +32-2-650-98-60. E-mail: henneghienbrait@europe.com

ISSN 1044-6672 print © 2002 Taylor & Francis Ltd
DOI: 10.1080/1044667031000137593

antibodies established in tertiary response of irradiated mice shows a reduced frequency of somatic mutation (Ismaïli *et al.*, 1999). The primary antibody repertoire seems frozen even in anamnestic responses.

The primary response to (4-hydroxy-3-nitrophenyl) acetyl (NP) in C57BL/6 mice has been extensively studied at both cellular and molecular levels (Allen *et al.*, 1987; Lalor *et al.*, 1992; McHeyzer-Williams *et al.*, 1993; Kelsoe, 1995; Nie *et al.*, 1997). At the serum level, NP binding antibodies are characterized by the expression of the λ1 light chain. High affinity NP binding IgGl are rapidly detected in the serum (8 days post immunization with NP-KLH). The heavy chain V regions are encoded by several germline segments of VH186.2/V3 subfamilly of the J558 family. A few days after NP-KLH injection, C57BL/6 mice develop splenic λ1+ foci and GC. Around 14 days post-immunization these λ1+ histological structures practically disappear. Among NP reactive germinal center B cells, the majority expresses BCR encoded by VH186.2-DFL16.2-JH2 rearrangements with canonical Tyr 95, a critical amino acid for NP binding. The VH186.2 GC B cells accumulate somatic point mutations including a recurrent point mutation in VH position 33 that replaces Trp with Leu. This mutation alone increases the affinity of VH186.2-DFL16.2-JH2/Vλ1 antibody by 10-fold indicating a selection of GC B cells into the higher affinity memory cell population.

The secondary NP response has been documented however, with complicated experimental designs that could bias the results (Cumano and Rajewsky, 1986; Blier and Bothwell, 1987; Siekevitz *et al.*, 1987; Decker *et al.*, 1995). Recently, Yi-Feng *et al.* (2000) I have examined bone marrow and splenic repertoire of NP specific anamnestic responses elicited in primed mice boosted with a low dose of soluble antigen. They identified a new and unexpected memory clonotype in which the VH-D segments were joined by glycine 95 instead of a tyrosine and devoid of the W33L point mutation. This observation suggests that primary and secondary responses are dominated by different clonotypes perhaps belonging to different B cell lineages.

Antibody molecules bearing kappa light chains have been also described in NP specific response (Mäkelä and Karjalainen, 1977; Reth *et al.*, 1978; Linton *et al.*, 1989). Some investigators observed that λ bearing B cells dominate primary responses, and secondary responses are dominated by κ bearing B cells. In our hands both λ and κ antibodies are detected during the anti-NP primary response but the serum levels of gk anti-NP appears to exceed the κ by 5 to 10-fold. This ratio is maintained in memory responses.

The dramatic alterations of repertoire detailed above in irradiated A/J mice led us to investigate the well defined NP primary response in irradiated and partially shielded C57BL/6 mice.

### RESULTS AND DISCUSSION

### Splenic T and B Cells are Significantly Reduced during the First Week Post Irradiation

Using flow cytometry we directly studied the effect of irradiation on T and B cell populations during the primary and secondary responses. Experiments made by Willems *et al.*, (1990) showed a splenic lymphopenia following mice irradiation and autoreconstitution. The different T and B cell populations were identified as CD3ε and B220 single positive cell, respectively. Nine days post irradiation (8 days after primary immunization) the FACS

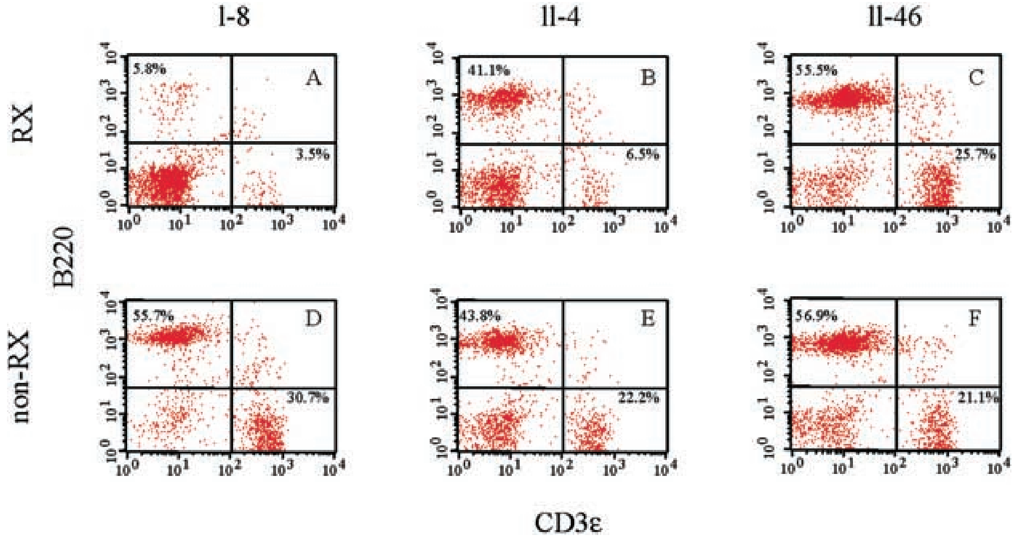

FIGURE 1 Reduction and reconstitution of B and T cell numbers in RX-P.S. following irradiation. Splenocytes were isolated from A–C, RX-P.S. and D–F, non-RX mice 8 days after primary immunization (A, D) with NP-KLH precipitated in Alum and 4 (B, E) and 46 days (C, F) after the second stimulation with NP-KLH in PBS. B220 and CD3ε single positive cell are defined as B cells and T cells, respectively.

staining indicated a dramatic decrease of B cell (6 vs. 56%) and T cell (4 vs. 31%) total number in RX-P.S. compared to non-RX mice, respectively (Fig. 1A, D). Nineteen days following irradiation (4 days after the second restimulation) RX-P.S. and non-RX mice had comparable B cell percentages (41 vs. 44%) but still differed in total T cell population (7 vs. 22%), respectively (Fig. 1B, E). Nevertheless RX-P.S. mice restored a similar T cell pool (21%) compared to non-RX-mice (26%) 61 days post irradiation (46 days after the second stimulation) (Fig. 1C,F). These results suggest that the B cell compartment reconstituted more rapidly than the T cell compartment following irradiation. These observations seem to indicate that T cells replenish slower than B cells.

### Reduction of Different B Cell Populations and Accumulation of Precursor Cells during the First Week Post Irradiation

Inasmuch as increasing sIgD expression is associated with B cell maturation, the experiments made by Allman *et al.* (1993) suggest HSA density is reciprocally correlated with B cell maturity. Moreover, they showed $HSA^{hi}$ splenic B cells predominate in early development as well as during initial stages of both adoptive bone marrow reconstitution and radiation-induced autoreconstitution. More importantly they demonstrate that $HSA^{hi}$ splenic B cell derived directly from $HSA^{hi}$ $sIg^{-}$ bone marrow progenitors. Using flow cytometry we defined in the spleen the B220 and HSA double positive cells as the total B cell compartment and $HSA^{hi}$ single positive cell as splenic precursor cells. Nine days after irradiation (8 days after primary immunization) we observed a 10-fold reduction in the different B cell populations in RX-P.S. (6%) compared to non-RX mice (56%) (Fig. 2A, B). The FACS analysis also demonstrated an enrichment of precursor cells in RX-P.S. (RX-P.S. 88%; non-RX 7%) (Fig. 2B). These results suggest that the splenic B cell lymphopenia in RX-P.S. mice is accompanied by precursor cell recruitment in order to reconstitute the B cell pool.

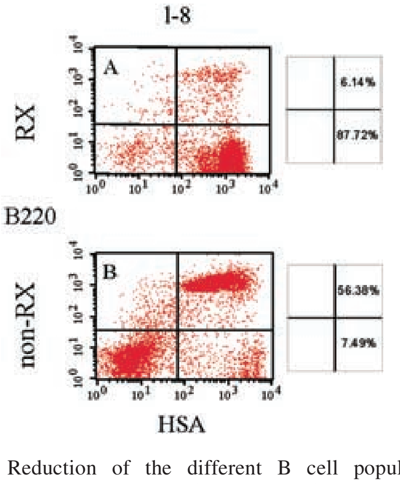

FIGURE 2 Reduction of the different B cell populations and accumulation of precursor cells in RX-P.S. following irradiation. Splenocytes were isolated from A, RX-P.S. and B, non-RX mice 8 days after primary immunization (A, B) with NP-KLH precipitated in Alum. B220 and HSA double positive and HSA high single-positive cells are defined to represent the different B cell populations and precursor cells, respectively.

### Splenic λ1+ Germinal Center Formation is Delayed but not λ1 Foci Pathway in Irradiated Mice

Splenic immunohistological analyses are well documented in the C57BL/6 primary NP response (Kelsoe, 1995 and see references herein). A few days after immunization, NP binding λ1+ GC are detected as well as extra follicular λ1+ foci. A kinetic analysis of the GC and foci was performed in irradiated and control mice at various times during the primary and secondary responses (Fig. 3). GC and follicles were visualized by staining with PNA and anti-IgD antibody, respectively. On serial sections, NP specific structures were defined as λ1+ cells.

Eight days post immunization, non-irradiated mice developed numerous GC (around 30 per section). Among these GC, 75% were λ1 + (Fig. 3U). λ1+ foci were still detectable (Fig. 3V). At day 14, foci disappeared and GC was highly reduced in number and size (fewer than 20 PNA+ cells per section). These observations are in agreement with previous published studies (Kelsoe, 1995).

During the first week after immunization, white pulp is reduced in irradiated mice. Rare small GC are identified (around five per section with fewer than 20 PNA+ cells) and few are λ1+. Nevertheless extrafollicular λ1 foci developed (Fig. 3A). At day 14, spleen pictures of irradiated mice were close to those of control mice at day 8. λ1+ GC (around 20 per section) and foci were well developed (Fig. 3B).

These observations suggest that in irradiated environment, GC formation is delayed (around one week) but foci pathway development appears less affected.

### Splenic λ1+ GC and Foci are Enlarged in Irradiated Mice during the Secondary Immune Response

The observations detailed above led us to investigate a secondary immune response in irradiated mice. 14 days after the first immunization, mice received an antigenic challenge in PBS. Spleens were collected 4 days later. Surprisingly irradiated mice had numerous enlarged λ1+ GC and foci as compared to control mice.

### Irradiated C57BL/6 Mice Produce Large Amount of NP Binding Antibodies

NP binding antibody levels (both IgM and IgG) were determined at day 14 in primary and secondary responses in a standard ELISA. (Fig. 4). Data represent mean ± SEM of serum binding curves on NPl7-BSA. Although B and T cells are reduced, RX-P.S. are able to mount an

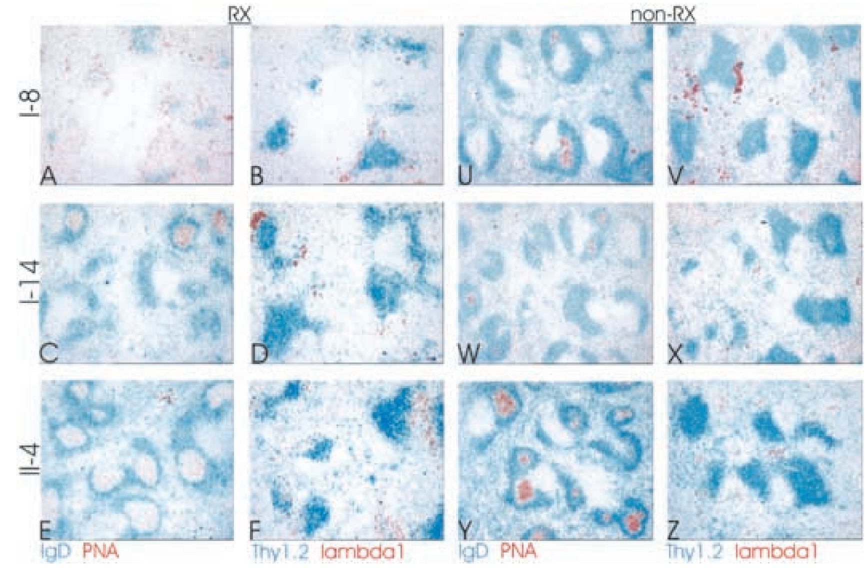

FIGURE 3 Effect of irradiation on splenic architecture and NP-specific structure development. Spleens from A–D, RX-P.S. and U–X, non-RX mice were collected 8 days (A, B, U and V) and 14 days (C, D, W and X) after primary immunization with NP-KLH precipitated in Alum. Spleens from E, F, RX-P.S. and Y, Z, non-RX mice were collected 4 days after the second stimulation with NP-KLH in PBS. GC and B cell follicles were visualized with PNA (red) and anti-IgD (blue) Ab, respectively (A, C, E, U, W and Y). In serial cryosections NP-specific structures and T cell zones (PALS) were visualized with anti-λ1 and anti-CD90.2 Abs, respectively (B, D, F, V, X and Z).

anti-NP response. This response increased after a second immunization. Surprisingly RX-P.S. produce higher NP binding antibody levels than control mice. Both anti-NP IgM and IgG are increased in these mice (data not shown). This unexpected observation has been poorly documented in literature.

We also measured the levels of lambda and kappa anti-NP antibodies (data not shown). We detected both isotype light chains but NP response is clearly dominated by λ molecules in both experimental groups after primary and secondary immunizations. The serum levels of κ anti-NP antibodies are very low, 10 to 15-fold less than levels of λ anti-NP antibodies. This ratio is maintained in both primary and secondary responses. Therefore, irradiated environment seems to favor NP specific response without altering the dominance of λ anti-NP molecules.

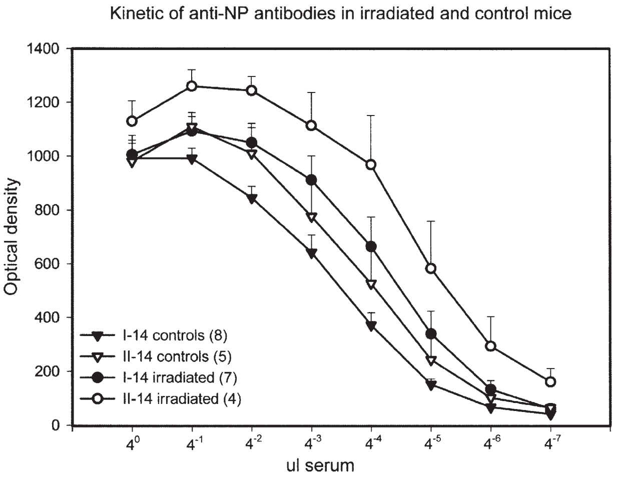

FIGURE 4 Increased anti-NP antibody response in RX-P.S. mice following irradiation. Sera from groups of 4 to 8 RX-P.S. (circle) and non-RX (triangle) mice immunized with NP-KLH precipitated in Alum were collected at day 14 (I-14, closed circle and triangle) and 14 days after the second stimulation with NP-KLH in PBS (II-14, open circle and triangle). Results represent mean ± SEM. Shown are Igs sera binding curves using $NP_{17}$-BSA. Number into brackets indicates number of mice.

### Irradiated C57BL/6 Mice Display an Impairment Affinity Maturation of NP Binding IgGl during Primary Response

NP primary response is characterized by the early synthesis of high affinity IgGl. Immunohistological and molecular analyses indicated that B cells producing these high affinity IgGl are first detected in GC. Our histological analyses demonstrated a delayed GC formation in RX-P.S., and raise the question of whether this delay was also associated with defects in affinity maturation. Thus we determined the relative affinity of the primary and the secondary responses using different NP-BSA substrates namely NP2-BSA and NP17-BSA. Both high and low affinity antibodies recognize NP17-BSA but only high affinity antibodies are able to bind on NP2-BSA. Results represent mean ± SEM of serum binding curves (Fig. 5). Close binding curves on NP2-BSA to NP17-BSA suggest a relative high affinity of serum IgGl. Fourteen days post immunization anti-NP high affinity IgGl were already detected in control group but these molecules failed to bind NP2-BSA in RX-P.S. Following a second immunization, irradiated recipients are able to synthesize anti-NP IgGl with increased affinity (Fig. 5). Therefore, we observed a transient decrease in serum affinity maturation probably due to a delay in GC reaction. Molecular analyses are required to define clonotypes used in primary and secondary immune responses and evaluate the frequency of somatic mutations.

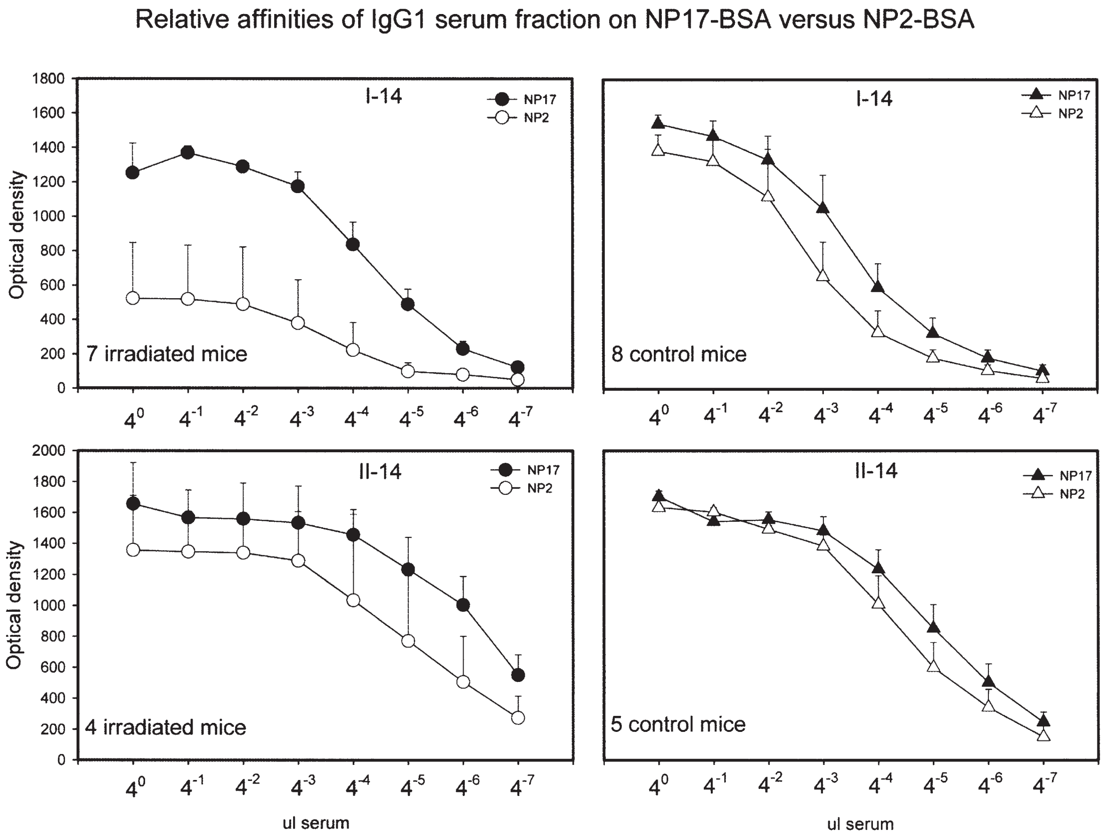

FIGURE 5 Altered affinity maturation process in RX-P.S. mice following irradiation. Sera from groups of 4 to 8 RX-P.S. (circle) and non-RX (triangle) mice immunized with NP-KLH precipitated in Alum were collected at day 14 (I-14) and 14 days after the second stimulation with NP-KLH in PBS (II-14). Results represent mean ± SEM. Shown are IgGl sera binding curves on $NP_{17}$-BSA vs. $NP_2$-BSA. Closed symbols represent binding curves on NP17-BSA and open symbols on NP2-BSA.

### CONCLUSIONS

FACS and immunohistochemical analyses indicate some splenic alterations as previously described in the irradiated A/J model. GC reaction is delayed (at least one week) although the kinetic of extrafollicular foci appearance is unchanged. Serological data indicate that irradiated C57BL/6 mice are able to mount a primary anti-NP response dominated by λ1 positive antibodies but fail to produce high affinity NP-binding IgGl antibodies. Following a second antigenic challenge, irradiated mice develop enlarged GC and foci. Furthermore, high affinity NP-binding IgG1 antibodies are detected.

Our observations are consistent with the following scenario. Reconstitution of lymphopenic environment favors the development of a first line of defense associated with marginal zone like B lymphocytes and plasma cells. The development of typical follicular B lymphocytes is only allowed after establishment of this first line (Agenes and Freitas, 1999; Martin *et al.*, 2002). This could explain the loss of CRIA idiotype in irradiated A/J mice and is compatible with the proposal that primary and secondary responses are mediated by different B cell subsets (Masungi Luko *et al.*, 2000). Likewise in the NP system, taking into account the recent data from the Cerny group (Yi-Feng *et al.*, 2000), we propose that reconstitution of irradiated C57BL/6 mice does not modify the primary repertoire but the appearance of a new clonotype (called the glycine 95 clonotype) may be severely delayed. This conclusion awaits for the molecular characterization of the NP repertoire in irradiated C57BL/6 mice.

### MATERIALS AND METHODS

### Mice

C57BL/6 mice were purchased from the Jackson laboratory (Bar Harbor, ME). Ten females 14-week- old were sublethally x-irradiated (750 rads) with their hind limbs covered with a lead shield (RX-P.S.) allowing autologous reconstitution by their own bone marrow stem cells.

### Immunization of Mice

Unirradiated C57BL/6 and irradiated mice (sex and age matched) were immunized by intra-peritoneal route (i.p.) with 50 μg of $NP_{36}$-KLH (Biosearch Technologies Inc., Novato, CA) precipitated in Alum (one day after irradiation) and challenged two weeks later with the antigen in saline. Mice were bled at weekly intervals.

### ELISA

The NP antibody levels were determined by ELISA. Briefly, 96 well microtiter plates were coated with 2 μg/ml of NP conjugated BSA (Biosearch Technologies Inc., Novato, CA) in PBS. Serial dilutions of sera were incubated at 4°C. Binding antibodies were detected by either POD-conjugated goat anti-mouse Igs (Sigma) or POD-conjugated monoclonal rat anti-mouse IgGl (LO-MGl-13, LO-IMEX, Brussels, Belgium).

The relative affinities of immune sera were measured by comparing their binding to differently haptenised carrier proteins ($NP_{17}$-BSA vs. $NP_2$-BSA). The $NP_{17}$-BSA ELISA detects low and high affinity antibodies and $NP_2$-BSA detects high affinity antibodies.

### Immunohistochemistry and Cytofluorometric Analyses

Eight and 14 days after the first vaccine and 4 days after the challenge vaccine, two or three mice per group were sacrificed. Spleens were removed and split in two parts. One part was embedded in OCT compound, frozen quickly in isopentane and stored at −80°C. Slices of 6–8 μm were made, de-embedded by washing in acetone for 10 min and transferred to PBS. The serial cryosections were stained for 60 min with the following FITC- or biotinylated-coupled antibodies (5 μg/ml) in PBS 1% blocking reagent (Boehringer Mannheim, Mannheim, Germany): PNA (peanut agglutinin, Sigma-Aldrich, Bornem, Belgium), anti-CD90.2 (53-2.1, BD Pharmingen, San Diego, CA, USA), anti-IgD (LO-MD-6, LO-IMEX, Brussels, Belgium) and anti-λ1 (MS40-13, kindly provided by P.-A. Cazenave and D. Rueff-Juy, Institut Pasteur, Paris, France). Tissues were then incubated for 30 min with either avidin-biotin-peroxydase complex (Vectastain ABC kit, Vector laboratories, Burlingame, CA) and anti-fluorescein-alkaline phosphatase Fab fragments (Roche Diagnostics, Brussels, Belgium) or avidin-biotin- alkaline phosphatase and anti-fluorescein-POD Fab fragments. Staining was finally developed with 3-amino-9-ethylcarbazole tablets (Sigma-Aldrich) and alkaline phosphates substrates (SK-5300, Vector Laboratories). Digitized images were captured using a Ikegarni CCD color camera (Ikegarni Tsushinki, Tokyo, Japan) ans analyzed using the Corel Draw seven software (Corel, Ottawa, Ontario, Canada).

Spleen cells from the other part were analyzed by flow cytometry with a FACSort Cytometer (BD Biosciences, Mountain View, CA). The cells were incubated with saturating dose of 2.4G2 (a rat anti-mouse FcR mAb; American Type Culture Collection (ATCC), Manassas, VA) for 10 min before staining to prevent Ab binding to FcR and were further stained with PE- or FITC-coupled mAbs including Ml/69 (anti-CD24), RA3-6B2 (anti-CD45R/B220), all from BD Pharmingen (San Diego, CA). Hamster mAb 145-2Cll to mouse CD3ϵ (Leo *et al.*, 1987) was purified and labeled in our laboratory. Cells were gated according to size and scatter to eliminate dead cells and debris from analysis.

*Acknowledgements*

This work is supported by the Belgian program on Interuniversity Poles of Attraction (P.A.I.) initiated by the Belgian State, Prime Minister's Office. The authors thank Dr Jan Cerny (Baltimore, USA) for helpful discussions.

### *References*

Agenes, F. and Freitas, A.A. (1999) "Transfer of small resting B cells into immunodeficient hosts results in the selection of a self-renewing activated B-cell population", *J. Exp. Med.* **189**, 319–330.

Allen, D.A., Cumano, A., Dildrop, R., Kocks, C., Rajewsky, K., Rajewsky, N., Roes, J., Sabliztky, F. and Siekevitz, M. (1987) "Timing, genetic requirements and functional consequences of somatic hypermutation during B-cell development", *Immunol. Rev.* **96**, 5–22.

Allman, D.M., Ferguson, S.E., Lentz, V.M. and Cancro, M.P. (1993) "Peripheral B cell maturation developmental intermediate in the production of long-lived marrow-derived B cells", *J. Immunol.* **151**, 4431–4444.

Blier, P.R. and Bothwell, A. (1987) "A limited number of B cell lineages generated the heterogeneity of a secondary immune response", *J. Immunol.* **139**, 3996–4006.

Cumano, A. and Rajewsky, K. (1986) "Clonal recruitment and somatic mutation in the generation of immunologic memory to the hapten NP", *EMBO J.* **5**, 2459–2468.

Decker, D.J., Linton, P.-J., Zaharevitz, S., Biery, M., Gingeras, T.R. and Klinman, N.R. (1995) "Defining subsets of naïve and memory B cells based on the ability of their progeny to somatically mutate *in vitro*", *Immunity* **2**, 195–203.

Ismaïli, J., Razanajaona, D., van Acker, A., Wuilmart, C., Mancini, I., Heinen, E., Leo, O., Lebecque, S., Urbain, J. and Brait, M. (1999) "Molecular and cellular basis of the altered immune response against arsonate in irradiated A/J mice autologously reconstituted", *Int. Immunol.* **11**, 1157–1167.

Kelsoe, G. (1995) "*In situ* studies of the germinal center reaction", *Adv. Immunol.* **60**, 267–288.

Lalor, P.A., Nossal, G.J.V., Sanderson, R.D. and McHeyzer-Williams, M.G. (1992) "Functional and molecular characterization of single (4-hydroxy-3-nitrophenyl)acetyl (NP)-specific, IgGl B cells from antibody-secreting and memory B cell pathway in the C57BL/6 immune response to NP", *Eur. J. Immunol.* **22**, 3001–3011.

Leo, O., Foo, M., Sachs, D.H., Samelson, L. and Bluestone, J.A. (1987) "Identification of a monoclonal antibody specific for murine T3 polypeptide", *Proc. Natl Acad. Sci. USA* **84**, 1374–1378.

Linton, P.-J., Decker, D.J. and Klinman, N.R. (1989) "Primary antibody-forming cells and secondary B cells are generated from separate precursor cell subpopulations", *Cell* **59**, 1049–1059.

Mäkelä, O. and Karjalainen, K. (1977) "Inherited immunoglobulin idiotypes of the mouse", *Immunol. Rev.* **34**, 119–138.

Manser, T., Wysocki, L.J., Margolies, M.N. and Gefter, M.L. (1987) "Evolution of antibody variable region structure during the immune response", *Immunol. Rev.* **96**, 141–162.

Martin, F. and Kearney, J.F. (2002) "Marginal-zone B cells", *Nat. Rev.-Immunol.* **2**, 323–335.

Masungi Luko, C., Vansanten, G., Ryelandt, M., Denis, O., Wuilmart, C., *et al.* (2000) "Distinct VH repertoires in primary and secondary B cell lymphocyte subsets in the preimmune repertoire of A/J mice: the CRIA idiotype is preferentially associated with the $HSA^{low}$ B cell subset", *Eur. J. Immunol.* **30**, 2312–2322.

McHeyzer-Williams, M.G., McLean, M.J., Lalor, P.A. and Nossal, G.J.V. (1993) "Antigen driving B cell differentiation *in vivo*", *J. Exp. Med.* **178**, 295–307.

Nie, X., Basu, S. and Cerny, J. (1997) "Immunization with immune complex alters the repertoire of antigen reactive B cells in the germinal centers", *Eur. J. Immunol.* **27**, 3517–3525.

Rathbun, G., Sanz, I., Meek, K., Tucker, P. and Capra, J.D. (1988) "The molecular genetics of the arsonate idiotypic system of A/J mice", *Adv. Immunol.* **42**, 141–162.

Reth, M., Hammerling, G.J. and Rajewsky, K. (1978) "Analysis of the repertoire of anti-NP antibodies in C57BL/6 mice by cell fusion. I. Characterization of antibody families in the primary and hyperimmune responses", *Eur. J. Immunol.* **8**, 393–400.

Siekevitz, M., Kocks, C., Rajewsky, K. and Dildrop, R. (1987) "Analysis of somatic mutation and class switching in naïve and memory B cells generating adoptive primary and secondary responses", *Cell* **48**, 757–770.

Vora, K.A., Tumas-Brundage, K.M. and Manser, T. (1998) "A periarteriolar lymphoid sheath-associated B cell focus response is not observed during the development of the anti-arsonate germinal center reaction", *J. Immunol.* **160**, 728–733.

Vora, K.A., Tumas-Brundage, K.M. and Manser, T. (1999) "Contrasting the *in situ* behavior of a memory B cell clone during primary and secondary immune responses", *J. Immunol.* **163**, 4315–4327.

Willems, F., Vansanten-Urbain, G., de Wit, D., Slaoui, M. and Urbain, J. (1990) "Loss of a major idiotype (CRIA) after repopulation of irradiated mice", *J. Immunol.* **144**, 1396–1403.

Yi-Feng, L.U., Singh, M. and Cerny, J. (2000) "Canonical germinal center B cells may not dominate the memory response to antigenic challenge", *Int. Immunol.* **13**, 643–655.

